# The Impact of Romantic Attachment Styles on Jealousy in Young Adults

**DOI:** 10.11621/pir.2023.0315

**Published:** 2023-09-30

**Authors:** Apollinaria V. Chursina

**Affiliations:** a Lomonosov Moscow State University, Moscow, Russia; b University of Granada, Spain; c Psychological Institute of the Russian Academy of Education, Moscow, Russia

**Keywords:** romantic attachment, romantic jealousy, two-dimensional model of attachment, attachment-related anxiety, attachment-related avoidance

## Abstract

**Background:**

Romantic attachment is reflected in various aspects of dyadic interaction in a couple, since it is a self-reinforcing system of cognitive, emotional and behavioral patterns. Romantic jealousy was shown to be associated with dimensions of attachment insecurity in various studies worldwide.

**Objectives:**

To identify differences in expressions of romantic jealousy based on romantic attachment style. To determine the influence of attachment-related anxiety and attachment-related avoidance on cognitive, emotional, and behavioral jealousy.

**Design:**

The sample comprised 171 heterosexual individuals. The “Experiences in Close Relationships — Revised” questionnaire (ECR-R; Fraley, Waller, & Brennan, 2000; adapted for Russian by Chursina, 2022) and “Multidimensional Jealousy Scale” (MJS; Pfeiffer, & Wong, 1989) were used.

**Results:**

A number of significant differences were identified between insecure and secure attachment styles. *Avoidant attachment* is characterized by cognitive jealousy, *ambivalent attachment* is characterized by cognitive and behavioral jealousy, while *dismissing attachment* showed no significant differences in the manifestations of jealousy in comparison with secure attachment style. Emotional jealousy is equally characteristic of all types. The primacy of romantic attachment in relation to cognitive and behavioral jealousy was also proved.

**Conclusion:**

The experience of jealousy differs among romantic attachment styles. *Attachment-related anxiety* is a predictor of intrusive thoughts and behavioral manifestations of jealousy, while *attachment-related avoidance* is less, the greater the manifestation of jealousy behaviors.

## Introduction

The concept of romantic attachment (Hazan & Shaver, 1987) goes back to the classical theory of attachment developed by J. Bowlby and M. Ainsworth (Ainsworth, Blehar, Waters, & Wall, 2015; Bowlby, 1982). This theory emphasized the importance of relationships with the primary caregiver for the child’s emotional development, and developed the idea of attachment as a complex system that includes cognitive, emotional, and behavioral components and patterns. The key notions are the internal working models of attachment, a system that includes unconscious attitudes and expectations about relationships in general. These ideas formed the basis of the two-dimensional model of individual differences in romantic attachment (Bartholomew, & Horowitz, 1991), where the main dimensions are image of the self and image of the significant other, later also identified as anxiety and avoidance of romantic attachment, respectively (Fraley, Waller, & Brennan, 2000).

These representations are mainly associated with evaluating oneself as deserving of love, support, and acceptance if the image of self scale or attachment-related anxiety is considered. Attachment-related avoidance, or image of the significant other, on the contrary, represents ideas about the people around as trustworthy and worthy of establishing close relationships. The combination of high and low values on these scales makes it possible to apply a typology of four romantic attachment styles: secure, fearful-avoidant (hereinafter “avoidant”), preoccupied (hereinafter “ambivalent”, since it corresponds to the classic ambivalent type described by J. Bowlby and M. Ainsworth), and dismissing-avoidant (hereinafter “dismissing”).

### Romantic Attachment Style as a Predictor of Relationship Functioning

Numerous studies by scientists around the world describe the relationship between romantic attachment style and different characteristics of dyadic interaction (e.g., Butzer, & Campbell, 2008; Conde, Figueiredo, & Bifulco, 2011). Since attachment is a self-reinforcing dynamic system, the characteristics of internal working models provide differences in the manifestations of interaction in a couple. Dimensions of attachment insecurity are associated with marital satisfaction (e.g., Rodriguez, Coy, & Hadden, 2021). Attachment-related avoidance prevents the development of intimacy and open communication in a couple, while attachment-related anxiety determines the need for confirmation of love from a partner. Modern research still debates whether marital satisfaction is higher if both partners have a secure attachment style, or whether it is enough for one of them to have it, thus acting as a buffer in the couple. However, recent research rejects the buffer hypothesis (Lozano, Sze, Fraley, & Chong, 2021). Nevertheless, in addition to anxiety and avoidance of romantic attachment, dyadic regulation processes are also important, for example, the context of interdependence and partners’ behavior in a particular situation (Overall & Simpson, 2015; Simpson, & Overall, 2014).

Various coping strategies can also serve as a buffer for the dimensions of attachment insecurity and relationship satisfaction. Emotion-focused dyadic coping buffers attachment-related anxiety, while problem-focused coping buffers avoidance (Vedelago, Balzarini, Fitzpatrick, & Muise, 2023). These ideas are consistent with the attachment security enhancement model (ASEM; Arriaga, Kumashiro, Simpson, & Overall, 2018). This approach is intended to neutralize the negative effects of insecure romantic attachment in a couple and to strengthen the model of the self and the model of the significant other. Attachment anxiety is addressed through increased self-confidence, while avoidance is reduced in situations of increased positive dependency.

Sexual functioning in a couple is also mediated by the influence of romantic attachment (Dunkley, Dang, Chang, & Gorzalka, 2016). Attachment-related avoidance has an overall, generalized negative impact on a couple’s sexual satisfaction (Heresi Milad, Rivera Ottenberger, & Huepe Artigas, 2014). Both attachment anxiety and avoidance are associated with negative emotional experiences during sex, although anxiety mediates caregiving motives at the same time, which promote positive emotional reactions (Beaulieu, Brassard, Bergeron, & Péloquin, 2022). Stefanou and McCabe (2012) also highlight the influence of both dimensions of attachment insecurity on sexuality in a couple, in particular, on satisfaction, frequency of sexual intercourse, and motivation, but emphasize the need for targeted research on the topic.

### Romantic Jealousy and Its Relationship with Romantic Attachment Style

Jealousy in romantic relationships is a complex of cognitive, emotional, and behavioral patterns that arise in response to a perceived threat to an individual’s relationship (Pfeiffer & Wong, 1989). The cognitive component of jealousy includes intrusive thoughts and suspicions about the potential infidelity of a romantic partner. The emotional aspect reflects the degree of negative affect regarding situations that provoke jealousy. Behavioral jealousy describes actual actions aimed at assuring a romantic partner’s fidelity. In this work, we rely specifically on a multidimensional approach to jealousy, since it is most consistent with the concept of the attachment system as a unity of emotional, cognitive, and behavioral patterns.

Scholars argue that there are different types of variables associated with jealousy. In their systematic review, Martínez-León et al. (2017) distinguish personal, interpersonal, and sociocultural variables. Despite the fact that there are ideas about the influence of cultural factors, physiological and hormonal processes, and a real experience of infidelity, a large body of research is associated precisely with personality traits, since they are clearly expressed in the processes of interpersonal interaction.

Jealousy in relationships is also related to romantic attachment style. Ambivalent attachment is most associated with various dimensions of jealousy (e.g., Marazziti, Consoli, Albanese, Laquidara, Baroni, & Catena Dell’osso, 2010), since it is characterized by a pronounced fear of rejection by the partner and represents a kind of insatiable need for love. Fear of losing the attachment figure contributes to increased patterns of jealousy. A similar pattern occurs with avoidant attachment, since both dimensions of attachment insecurity are present in this style. The difference lies in the degree of involvement in close relationships against the background of pronounced jealousy. People with anxious attachment describe themselves as being more jealous and having low self-esteem.

Individuals with ambivalent attachment have significantly higher levels of jealousy, as well as fewer positive feelings and lower self-esteem in laboratory-induced jealousy situations (Kim, Feeney, & Jakubiak, 2018). In experimental conditions, it was individuals with anxious attachment who demonstrated a greater number of jealousy patterns. There are also gender differences in jealousy (e.g., Güçlü, Şenormancı, Şenormancı, Köktürk, 2017), with women exhibiting emotional and cognitive jealousy to a greater extent.

Jealousy demonstrates age differences in women (Shackelford et al., 2004). Consequently, it could also be emphasized that jealousy decreases as a marriage progresses. Although this could be explained by aging and hormonal changes, we also emphasize that the dimensions of attachment insecurity change as the marriage progresses (Fraley, 2019). A person accumulates the experience of positive interaction in a stable relationship with a romantic partner, which helps reduce attachment anxiety.

Therefore, there are differences in the expression of jealousy depending on the romantic attachment style (Sharpsteen, & Kirkpatrick, 1997). Internal working models of attachment provide a certain scheme, a predisposition regarding the perception of relationships as a whole and, accordingly, reinforce their functioning in a certain way. Jealousy, since it represents some anticipation of potential infidelity and threat to the relationship, is also a kind of internal working model, so it seems necessary to study the characteristics of this associations for different romantic attachment styles.

### The Current Study

The purpose of this study is to replicate existing research in the field of psychology of attachment and romantic jealousy for the first time with a Russian sample. The study is designed to expand existing understanding of the relationship between attachment style and romantic jealousy and to identify characteristics specific to different attachment styles.

## Methods

### Participants

The sample comprised 171 heterosexual persons aged 20–54 (M = 33.06, SD = 7.94), 64 men (37.43%) and 107 women (62.57%). All the participants cohabited with their romantic partners. 77.2% had higher education, 10.5% had incomplete higher education, 2.3 and 9.9% had completed secondary and secondary specialized education, respectively.

### Instruments

*The Experiences in Close Relationships - Revised questionnaire* (ECR-R; Fraley, Waller, & Brennan, 2000; adapted for Russian by Chursina, 2022) was used to assess romantic attachment style. It is based on a two-dimensional model of individual differences in romantic attachment that assesses attachment-related anxiety and attachment-related avoidance. The questionnaire consists of 36 statements related to romantic relationships, rated on a 7-point Likert scale of agreement/disagreement, and is aimed at determining the degree of anxiety and avoidance of intimacy in relationships with a romantic partner.

*The Multidimensional Jealousy Scale* (MJS; Pfeiffer, & Wong, 1989) was used to evaluate the patterns of romantic jealousy considering its three types of patterns (emotional, cognitive, and behavioral patterns). It consists of 24 statements related to various aspects of jealousy, rated on a 7-point Likert scale based on frequency of manifestations for the cognitive and behavioral scales, and by severity of manifestations for the emotional scale.

### Procedure

All study participants were informed about its objectives and gave voluntary informed consent to participate. Data was collected online.

## Results

### Relationship Between the Indicators of Romantic Attachment and Jealousy

In a study of jealousy in adults with different romantic attachment styles, significant correlations were identified between the scores on the multidimensional jealousy scale and measures of insecure romantic attachment. Correlations were found between the cognitive jealousy scale and both dimensions of romantic attachment insecurity: anxiety (*r* = 0.50, *p* < 0.00) and avoidance (*r* = 0.29, *p* < 0.00), as well as with the type of romantic attachment variable (*r* = 0.21, *p* < 0.01). Romantic attachment anxiety also showed a significant correlation with behavioral jealousy (*r* = 0.41, *p* < 0.00).

**Table 1:** Descriptive statistics for MJS questionnaire scales in individuals with different romantic attachment styles

Scale	Secure attachment style		Avoidant attachment style		Ambivalent attachment style		Dismissing attachment style	
	M	SD	M	SD	M	SD	M	SD
Cognitive jealousy	2.11	1.25	3.38	1.39	3.24	1.73	2.37	1.15
Emotional jealousy	4.88	1.12	5.12	1.13	5.01	1.16	5.03	0.90
Behavioral jealousy	2.07	0.86	2.75	1.19	2.77	1.06	1.95	0.80

Therefore, adults diagnosed with attachment insecurity are more prone to demonstrate cognitive jealousy, namely, to have obsessive thoughts about the potential infidelity of their partner, while the correlation of this parameter is significantly higher for the measure of attachment anxiety than for avoidance. Behavioral jealousy (committing acts driven by jealousy and aimed at testing hypotheses about a partner’s potential infidelity) is characteristic of adults with romantic attachment anxiety.

### Jealousy Manifestations in Individuals with Different Romantic Attachment styles

To identify more specific patterns of manifestations of jealousy in adults with different types of romantic attachment, we applied one-way analysis of variance, namely the method of multiple comparisons (Tukey HSD test).

#### Comparison of Secure and Avoidant Romantic Attachment Styles

Two significant differences were identified: on the cognitive jealousy scale (HSD = –1.27, *p* < 0.00) and on the behavioral jealousy scale (HSD = –0.68, *p* < 0.003). Securely attached individuals have significantly fewer intrusive jealous thoughts about potential infidelity in its various forms than those with an avoidant attachment to the romantic partner. Additionally, securely attached individuals exhibit significantly fewer jealousy behaviors than avoidantly attached adults. A negative self-image and a negative image of a significant other represent “double doubt” and provoke jealousy in its various dimensions: the person doubts their own worthiness for love and at the same time doubts their partner and their partner’s positive qualities.

#### Comparison of Individuals with Secure and Ambivalent Romantic Attachment Styles

Two significant differences were identified: on the cognitive jealousy scale (HSD = –1.13, *p* < 0.01) and on the behavioral jealousy scale (HSD = –0.70, *p* < 0.03). Individuals with ambivalent attachment to the romantic partner demonstrate significantly more behavioral manifestations of jealousy and have significantly more thoughts about the possible infidelity of the partner than adults with secure attachment. In this case, the main role is played by the negative image of self, where people consider themselves undeserving of love, support, and acceptance, which corresponds to the classical ideas of attachment theory.

#### Comparison of Individuals with Secure and Dismissive Romantic Attachment Styles

No significant differences were found.

The study found a number of significant differences in the expression of jealousy among young adults with different romantic attachment styles. These differences, revealed through multiple comparisons, where jealousy is significantly higher in individuals with avoidant and ambivalent attachment—that is, where insecurity is manifested through anxiety in romantic attachment—turned out to be more congruent with classical ideas about the relationship between romantic attachment and jealousy. The fact that rejecting romantic attachment (and this is the type where insecurity is associated only with the avoidance dimension, against the background of low romantic attachment anxiety) did not find significant differences compared to secure attachment only confirms that the avoidance dimension cannot be a factor associated with the origin of jealousy, to confirm which we used regression analysis methods (linear regression).

### The Impact of Romantic Attachment on Jealousy Patterns

Three models were built, one for each dimension of jealousy (cognitive, emotional, behavioral), where the independent variables were both dimensions of attachment insecurity, anxiety and avoidance.

The model of cognitive jealousy (*R*2 = 0.25, F = 28.17, *p* < 0.00), explained through measures of attachment insecurity, revealed romantic attachment anxiety as a significant predictor (β = 0.49, *t* = 6.61, *p* < 0.00). Therefore, anxiety in romantic attachment is a prerequisite for the formation of the cognitive aspect of jealousy, namely jealous thoughts about one’s partner.

The model of emotional jealousy explained through indicators of romantic attachment insecurity was statistically insignificant (*R*2 = 0.01, F = 0.89, *p* < 0.41). Therefore, negative emotional patterns during the experience of jealousy are not conditioned by romantic attachment, that is, in this case we can say that all people equally negatively experience situations that provoke jealousy.

The model of behavioral jealousy (*R*2 = 0.27, F = 31.13, *p* < 0.00), explained through indicators of insecurity of romantic attachment, showed that both anxiety (β = 0.57, *t* = 7.78, *p* < 0.00) and avoidance (β = –0.15, *t* = –2.11, *p* < 0.04) are significant prerequisites for the development of behavioral jealousy. Therefore, romantic attachment anxiety shapes patterns of behavioral jealousy, that is, those actions that are associated with the experience of jealousy or testing a romantic partner for potential infidelity. However, avoidance, on the contrary, inversely predicts jealous behavior, that is, less avoidance is a prerequisite for jealous behavior.

## Discussion

This research demonstrated that the experience of jealousy differs for adults with different romantic attachment styles. A number of differences were identified between insecure romantic attachment styles compared to secure attachment styles. Avoidant attachment is characterized by cognitive jealousy (thoughts about a partner’s potential infidelity); such individuals are both emotionally sensitive and suspicious. Ambivalent attachment is characterized by both cognitive and behavioral jealousy (specific actions with the aim of preventing or localizing the threat of infidelity of a partner), which corresponds to the theoretical idea that people with this type of attachment are the most jealous. It is noteworthy that dismissing attachment showed no fundamental differences in the manifestations of jealousy in comparison with secure attachment. At the same time, emotional jealousy is characteristic of all attachment styles equally, demonstrating that the perceived threat of infidelity equally causes negative emotions in each individual.

Other studies (e.g., Rydell, & Bringle, 2007) highlight the association of attachment anxiety with cognitive and behavioral jealousy, but not with emotional jealousy. We believe this is because, regardless of attachment style, monogamous relationships still involve exclusivity, meaning that potential infidelity will trigger negative emotional states. However, some scholars have found connections between attachment anxiety and all components of jealousy (e.g., Rodriguez, DiBello, Øverup, & Neighbors, 2015). Moreover, there is evidence supporting the association of attachment anxiety with emotional, cognitive, and behavioral online jealousy (Sullivan, 2021).

The primacy of romantic attachment in relation to cognitive and behavioral jealousy was also described in the present study: it is the anxiety of attachment to a romantic partner that is a significant prerequisite for their development. Our findings are consistent with the results of Deng et al. (2023), who noted the predictive effect of attachment anxiety in relation to jealousy. This connection is mediated by a low level of self-differentiation. Indeed, attachment anxiety is associated with a state of alertness and control (like a “radar”), and fear of rejection. Neuroticism (Richter, Schlegel, Thomas, & Troche, 2022) and low self-esteem (DiBello, Rodriguez, Hadden, & Neighbors, 2015) were also reported as predictors of romantic jealousy; therefore, more research on romantic jealousy, attachment, and personality traits is needed. In addition to being significantly associated with various aspects of jealousy, attachment-related anxiety is also associated with dyadic interactions, for example, dyadic distrust (Toplu-Demirtas, Akcabozan-Kayabol, Araci-Iyiaydin, & Fincham, 2022). Furthermore, anxiously attached individuals induce feelings of guilt in their partner in response to negative situations in the relationship, and thus feel more secure and stable (Overall, Girme, Lemay, & Hammond, 2014).

It is advisable to study dyadic mechanisms in the manifestation of jealousy depending on the style of romantic attachment. Pfaus et al. (2023) consider ideas about synchrony in relationships, including in relation to the attachment system in a couple. Since attachment is self-reinforcing in both caregiver and romantic partner relationships, it will inevitably influence various aspects of relationship quality by reinforcing dysfunctional patterns due to the lack of positive experiences. Depending on the patterns of attachment in a couple, behavioral and emotional synchrony or asynchrony may occur, which is also associated with hormonal processes. Moreover, perceptions across dyads of attachment insecurity are consistent (Molero, Shaver, Fernandez, Alonso-Arbiol, & Recio, 2016) and are associated with low relationship satisfaction, so the consistency of perceptions of jealousy patterns remains an issue.

## Conclusion

The study demonstrated the strong association and primacy of romantic attachment in relation to jealousy. These findings are of particular interest in the context of attachment theory in general and the two-dimensional model of romantic attachment in particular. First, we established the patterns of the experience of jealousy within a multidimensional model of jealousy and romantic attachment: we found that manifestations of cognitive and behavioral jealousy vary for different types of romantic attachment; in particular, these phenomena are most characteristic of individuals with avoidant and ambivalent attachment. Second, we found that the anxiety of romantic attachment acts as a predictor of thoughts and behavioral manifestations of jealousy, while there is less avoidance of romantic attachment, the greater the manifestation of jealousy behaviors.

## Limitations and Suggestions for Future Research

Several limitations must be taken into account before interpreting these results. First of all, the study was conducted on a sample where the vast majority of participants have higher education and belong to the middle class, living mainly in Moscow. Despite the particular features of the education system in Russia, its universal accessibility at all levels, the study should be expanded to other social contexts and regions, since the results may differ. The sample was also not age-balanced. In addition, in view of the fact that the supposition of continuity of adult and child attachment, strictly speaking, is an assumption and has been confirmed by only a few longitudinal studies, it is difficult to unequivocally state the primacy of romantic attachment in relation to jealousy. Finally, it is also necessary to examine the influence of both personality characteristics potentially associated with jealousy and attachment style (for example, the level of self-differentiation or paranoid traits), as well as to examine the processes of dyadic interaction, since this study involved individuals, but not couples.
